# Primary uterine broad ligament ependymoma with *CHEK2* *p.H371Y* germline mutation: A CARE‐compliant case report uterine broad ligament ependymoma

**DOI:** 10.1111/jog.15065

**Published:** 2021-10-30

**Authors:** Jiaxin Yin, Min Yao, Hongdi Lu, Xiaofeng Cong, Pengfei Cui, Ziling Liu

**Affiliations:** ^1^ Department of Oncology The First Hospital of Jilin University Jilin China; ^2^ Department of Pathology The Second Hospital of Jilin University Jilin China; ^3^ Department of Oncology Jilin Cancer Hospital Jilin China

**Keywords:** *CHEK2 p.H371Y*, ependymoma, next‐generation sequencing, uterine broad ligament

## Abstract

Ependymomas arise from ependymal cells lining the ventricles and central canal of the spinal cord and can occur throughout the whole neuraxis. The lesion rarely occurs in extracranial or extraspinal regions, particularly in the uterine broad ligament. Thus, for the pathogenesis of nonsacral extra‐central nervous system (CNS) ependymomas remains elusive. Here, we describe a rare case of primary uterine broad ligament. ependymoma with cell‐cycle‐checkpoint kinase 2 (*CHEK2*) *p.H371Y* germline mutation. A 45‐year‐old woman presented with a uterine mass. The transvaginal sonographic examination confirmed a 4.4 cm × 3.7 cm, cystic and solid, mass located on the right side uterine wall near isthmus. First, laparoscopy with the neoplasm resection was carried out. Based on morphological and immunohistochemical characteristics of tumor cells that expressed glial fibrillary acidic protein (*GFAP*), *S‐100*, and vimentin, the tumor was diagnosed as an ependymoma. After that, she underwent a laparotomic total hysterectomy, bilateral salpingo‐oophorectomy, and lymphadenectomy. Furthermore, we performed next‐generation sequencing (NGS) of the patient's resected tumor tissue and peripheral blood and identified a novel *CHEK2 p.H371Y* germline mutation. Following surgery, the patient received oral tamoxifen (10 mg 2/day) and followed by letrozole (2.5 mg/day) for 6 months. The patient remained disease‐free after 4 years of follow‐up. Conceivably, *CHEK2 p.H371Y* is a driving gene for the development of extra‐CNS ependymoma.

## Introduction

Ependymomas predominantly develop from neuroectodermal organs. Primary extraneural ependymomas are rare and typically defined as a sacrococcygeal, pelvic, or extrapelvic ependymoma based on tumor location. The most common site for extraneural ependymomas remains the sacrococcygeal region, with more than 50 reported cases.^1^ However, thus far, the pathogenesis of nonsacral extra‐central nervous system (CNS) ependymomas remains elusive. Here, we report a rare case of primary uterine broad ligament ependymoma with cell‐cycle‐checkpoint kinase 2 (*CHEK2*, also known as *CHK2*) *p.H371Y* germline mutation. The patient was treated with surgery, followed by endocrine therapy.

## Case Report

**FIGURE 1:**
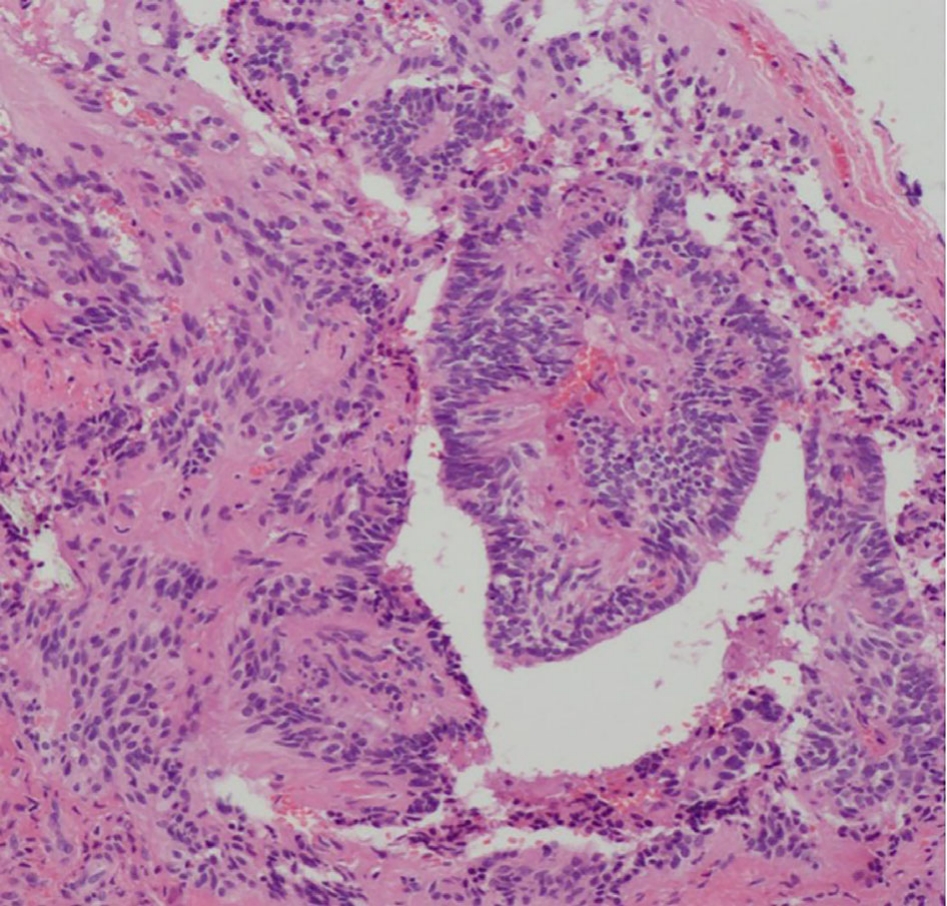
Histopathological examination revealed perivascular pseudorosettes and ependymal rosettes, with a central lumen radially surrounded by tumor cells (hematoxylin and eosin [H&E] stain, ×100 magnification)

**FIGURE 2:**
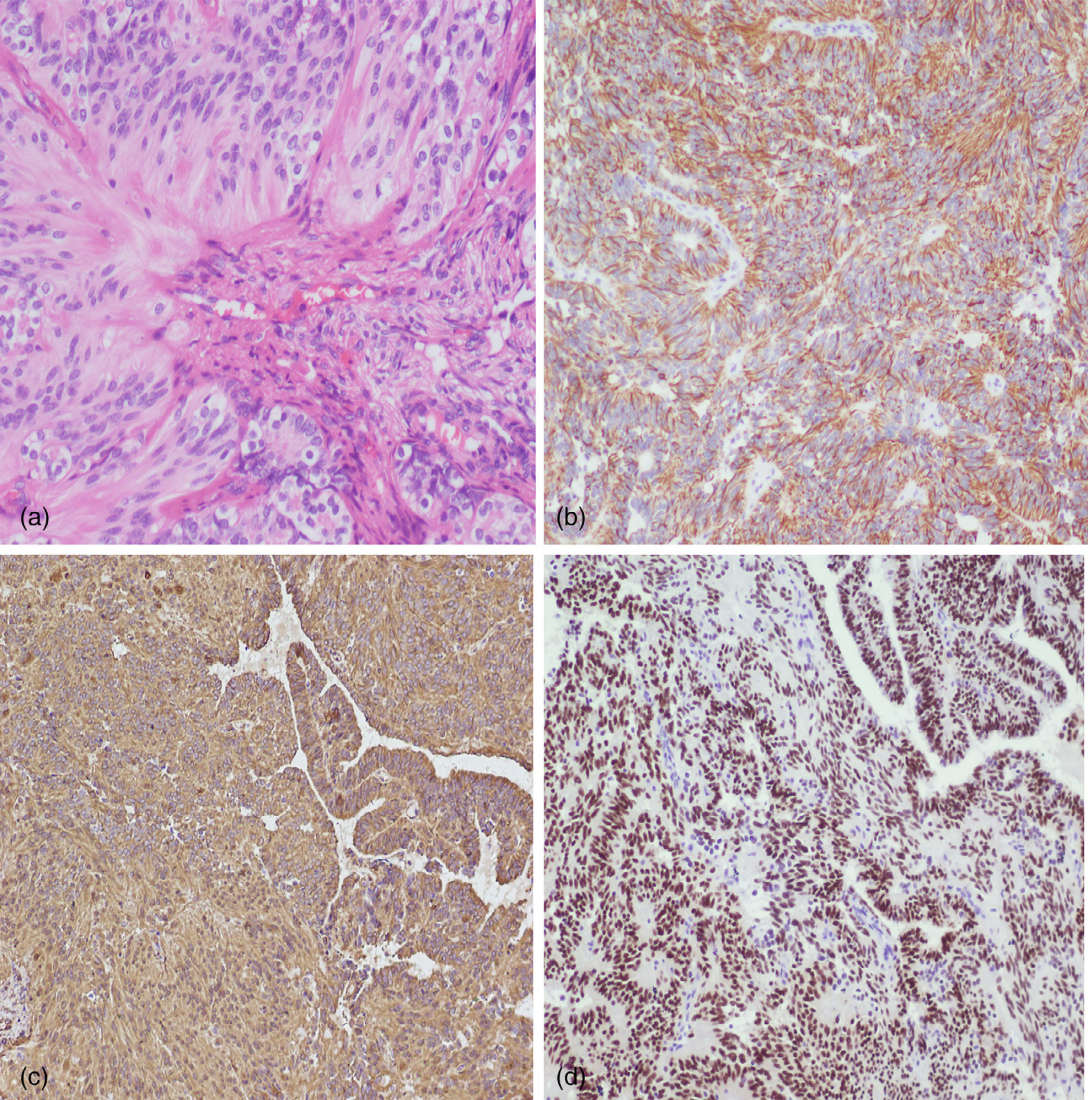
(a) Histopathological examination revealed perivascular pseudorosettes and ependymal rosettes, with a central lumen radially surrounded by tumor cells (hematoxylin and eosin [H&E] stain, ×200 magnification). (b) Tumor cells showing diffuse cytoplasmic positivity with glial fibrillary acidic protein (immunohistochemistry; glial fibrillary astrocytic protein ×100). (c) Tumor cells showing cytoplasmic positivity with vimentin (immunohistochemistry; vimentin ×100). (d) Tumor cells showing nuclear positivity with paired box 8 (immunohistochemistry; paired box 8 × 100, by EnVision)

A 45‐year‐old woman presented with chronic multilevel spinal pain. Transvaginal sonographic examination revealed a 4.4 cm × 3.7 cm, cystic and solid, mass located in the right side of the uterine wall near isthmus. She underwent laparoscopy with the neoplasm resection. At the first time of surgery, a uterine mass of approximately 4.0 cm × 4.0 cm was seen protruding toward the right broad ligament, and the degree of bulging did not exceed the right round ligament. Grossly, tumor presented as a cystic and solid, gray section, crisp, and rotting meat‐liked lesion (Figure S1). Based on both histopathological and immunohistochemical examination of the mass, a diagnosis of ependymoma was made. Subsequently, after 7 days, she underwent a laparotomic total hysterectomy, bilateral salpingo‐oophorectomy, and pelvic lymphadenectomy. Furthermore, gross pathological findings revealed that tumor exhibited infiltrated right myometrium and parametrial tissue, right fallopian tube wall, and surrounding tissue, and there were two metastatic obturator lymph nodes. The other pelvic organs and abdomen appeared normal. At high‐power magnification of the specimen, tumor cells were consistent in size and arranged densely. Tumor cells exhibited uniform oval to the spindle, hyperchromatic nuclei, and lightly stained cytoplasm. Furthermore, tumor specimens revealed perivascular pseudorosettes and ependymal rosettes, with a central lumen radially surrounded by tumor cells, a structure similar to the medullary central canal (Figure 1). Immunohistochemical staining revealed that the tumor cells were diffusely immunoreactive to *GFAP*, vimentin, *PAX‐8*, S‐100 protein, P16, epithelial membrane antigen, and AE1/AE3. Among them, *GFAP*, *S‐100*, vimentin expression is characteristic of the tumor (Figure 2). Immunostaining for hormone receptors showed positive staining for progesterone receptor (*PR*) and estrogen receptor (*ER*) in many tumor cells. Negative staining for P53, calretinin, a‐inhibin, cytokeratins 7 and CD10 was observed. Postoperative positron emission tomography and computed tomography (PET/CT) scans confirmed no residual lesions and distant metastasis. Furthermore, using PCR and NGS, a novel *CHEK2 p.H371Y* germline mutation, which specifically referred to H371Y hybrid germline mutation caused by the single base substitution of exon 11 of *CHEK2* gene, was detected in the DNA obtained from tumor tissue and DNA peripheral blood (Figure S2). Hormonal therapy with an aromatase inhibitor or chemotherapy was considered most effective and the patient opted for endocrine therapy. She received oral tamoxifen (10 mg twice/day) followed by letrozole (2.5 mg/day) for 6 months. Follow‐up CT scans of the chest, abdomen, and pelvis showed no evidence of disease. She remained asymptomatic and disease‐free during the follow‐up of 4 years.

Informed written consent was obtained from the patient for publication of this case report and accompanying images.

## Discussion

Primary uterine broad ligament ependymoma is an extremely rare extra CNS neoplasm with only seven previously reported cases^2^, ^3^, ^4^, ^5^, ^6^, ^7^ in the literature worldwide. Of these, four tumors originated in broad ligament,^2^, ^3^, ^4^ one in uterosacral ligament,^5^ one in mesovarium right ovary,^6^ and one in endometrium^7^ (Table 1). Ependymomas predominantly affect women of relatively younger age (mean age, 38 years, 13–61 years). The extra‐CNS ependymoma is usually large, partially cystic, and solid masses (mean size, 9 cm, 1–14 cm) that cause patients to experience abdominal pain. The solid mass was mostly associated with hemorrhage and necrosis. Tumors were located mostly at the right (six of eight cases) compared with the left (one of eight cases). Histopathologically, only two ependymomas exhibited myxopapillary, and the others presented the classical morphology. Immunohistochemically, the diagnosis of typical primary extra‐CNS ependymoma was supported by the presence of ependymal rosettes, perivascular pseudorosettes, and *GFAP* immune‐positivity. In most cases, extra‐CNS ependymoma is histologically classified as low‐grade ependymomas; however, they can be potentially fatal. Notably, of histologically classified low malignant tumors, nearly 50% of tumors have been recognized to exhibit a metastasis at initial treatment. Furthermore, ependymoma cells metastasize to and recur mostly in the peritoneum. Perhaps, as there is no blood–brain barrier impeding dissemination, the tumor cells can easily access to the bloodstream and lymphatic system. The findings were consistent with that of the previously reported cases, where the subcutaneous sacrococcygeal ependymoma is as high as 20% with distant metastasis.^8^

**TABLE 1:** Clinicopathological characteristics of reported ependymomas of the uterus or uterine ligaments

References	Age	Location	Symptom	Size (cm)	Gross appearance	Histological type	Metastasis	Therapy	Recurrence	Follow up
^2^	27	Right broad ligament	Lower abdominal pain	14	Cystic and solid	Classical	Omentum	Surgery + chemoradiotherapy		5 months
^3^	22	Left broad ligament	Left adnexa mass	7	Cystic	Myxopapillary	None	Surgery + chemotherapy		18 months
^4^	13	Right broad ligament	Pelvic mass	12	Cystic	Classical	None	Surgery	Retroperitoneum (11 years 24 years), surgery	26 years
^4^	45	Right broad ligament	Lower abdominal pain	13	Hemorrhagic necrotic	Classical	Bladder	Surgery + radiotherapy		21 months
^5^	48	Right uterosacral ligament	Suprapubic discomfort	11	Cystic and solid	Classical	Peritoneal	Surgery + radiotherapy		18 months
^6^	47	Mesovarium right ovary	Suprapubic discomfort	1	Solid	Classical		Surgery		2 years
^7^	61	Endometrium			Solid	Papillary	Abdomen	Surgery		
Current case	45	Right broad ligament	Uterine mass	4	Cystic and solid	Classical	Obturator lymph node	Surgery + endocrimotherapy		4 years

Because of the rarity of this neoplasm, no standard or overall effective treatment or chemotherapy has been identified. Previously, most of the patients underwent surgery at initial treatment, followed by adjuvant therapy after surgery, including chemotherapy, radiotherapy, chemoradiotherapy, or endocrine therapy. The patient exhibited recurrence at 11 and 24 years, postoperatively.^4^ Nevertheless, surgical resection may be most effective when the recurrent tumor is detected. Although data regarding survival remains limited, a few of the cases exhibited prolonged survival and slow growth of the tumor, even in the presence of metastasis.

While the female gender biases of sacrococcygeal tumors are similar to that of CNS ependymomas, cases of extra‐axial ependymomas arising elsewhere appear to occur exclusively in women.^9^ Ependymomas arise throughout the neuraxis and have an intimate relationship to ependymal cells or their remnants. However, the pathogenesis of nonsacral extra‐CNS ependymomas remains largely unclear. Ependymomas in the ovary, the uterine ligaments, or the mediastinum have been described very rarely. This can be attributed to the fact that ependymomas in the ovary, broad ligament, and mediastinum might originate from neuroectodermal teratoma tissue.^10^ Besides, according to another theory, under the influence of female hormones, misdirected primordial germ cells transform to ependymal cells, and this could possibly explain the female predominance of such tumors.^9^ It might also explain why extra‐CNS ependymomas stain strongly and diffusely positive for estrogen and progesterone receptors.

NGS of DNA obtained from peripheral blood and tumor tissues samples revealed that *CHEK2 p.H371Y* germline mutations caused by single base permutation in exon 11. This finding is unprecedented. *CHEK2* is located on the long (q) arm of chromosome 22. *CHEK2* is a tumor suppressor gene that encodes a multifunctional kinase that is activated essentially by the ataxia telangiectasia‐mutated (ATM) protein in response to DNA double‐strand breaks.^11^ Activated *CHEK2*, in turn, phosphorylates several critical cell‐cycle proteins, including p53, Cdc25, and *BRCA1*, which trigger cell‐cycle arrest, apoptosis, and the activation of DNA repair.^12^, ^13^, ^14^ *CHEK2 p.H371Y* mutation is located within the activation loop of the *CHEK2* protein kinase domain, which is crucial for the activation of *CHEK2* in response to DNA damage. Further functional analysis revealed that the *CHEK2 p.H371Y* mutation causes a dramatic decline in CHEK2 activity and is a pathogenic mutation. *CHEK2 p.H371Y* confers a 2.43‐fold increase in breast cancer risk among Chinese women.^15^ Thus, *CHEK2 p.H371Y* mutation may share some similarity to *BRCA1* mutation; therefore, carriers of *CHEK2 H371Y* mutation may be considered potential candidates for treatment with poly ADP‐ribose polymerase‐1 inhibitors.^16^

The finding of *CHEK2 p.H371Y* mutation in extra‐CNS is novel to the molecular profile of CNS ependymoma. However, due to limited tissue availability and lack of funding for more in‐depth molecular testing, whether *CHEK2 p.H371Y* is a pathogenic gene for the development of extra‐CNS ependymoma is currently uninvestigated. Thus, future studies are needed to confirm the gene expression profile of extra‐CNS ependymomas, which may provide insight into the cellular origin and molecular drivers of these rare tumors. In summary, primary uterine broad ligament ependymomas are extremely rare kinds of extra central nervous system ependymomas and there is no one unified standard treatment for this. Thus, to better understand the molecular pathogenesis of this rare neoplasm, further molecular profiling studies are needed to guide clinical therapy. The finding of *CHEK2 p.H371Y* might conceivably become a major breakthrough for the treatment of extra‐CNS ependymomas.

## Conflict of Interest

None declared.

## Author Contributions

Jiaxin Yin was mainly responsible for drafting of the manuscript and revising. Min Yao was responsible for supporting pathology images. Xiaofeng Cong was responsible for acquisition of data and follow up. Hongdi Lu contributed analysis and Interpretation of data. Pengfei Cui contributed supervision. Ziling Liu contributed conception and design.

## Supporting information


**Figure S1** Macroscopic photo of this neoplasm

**Figure S2** Cell‐cycle‐checkpoint kinase 2 (*CHEK2*) is located on the long (q) arm of chromosome 22. *p.H371Y* germline mutation is caused by single base permutation in exon 11 of the *CHEK2* gene.

## Data Availability

The data that support the findings of this study are available from the corresponding author upon reasonable request.
