# Supplementary figures and images for: Primary uterine broad ligament ependymoma with CHEK2 p.H371Y germline mutation: A CARE‐compliant case report uterine broad ligament ependymoma
Source: J Obstet Gynaecol Res. 2021 Oct 30;48(1):266–70. doi: 10.1111/jog.15065 (PMC9298396; doi:10.1111/jog.15065)

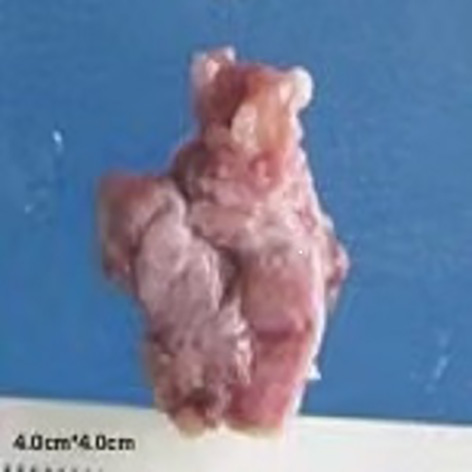

Supplement: Supplementary file 1 — Figure S1 Macroscopic photo of this neoplasm [file JOG-48-266-s002.jpg]

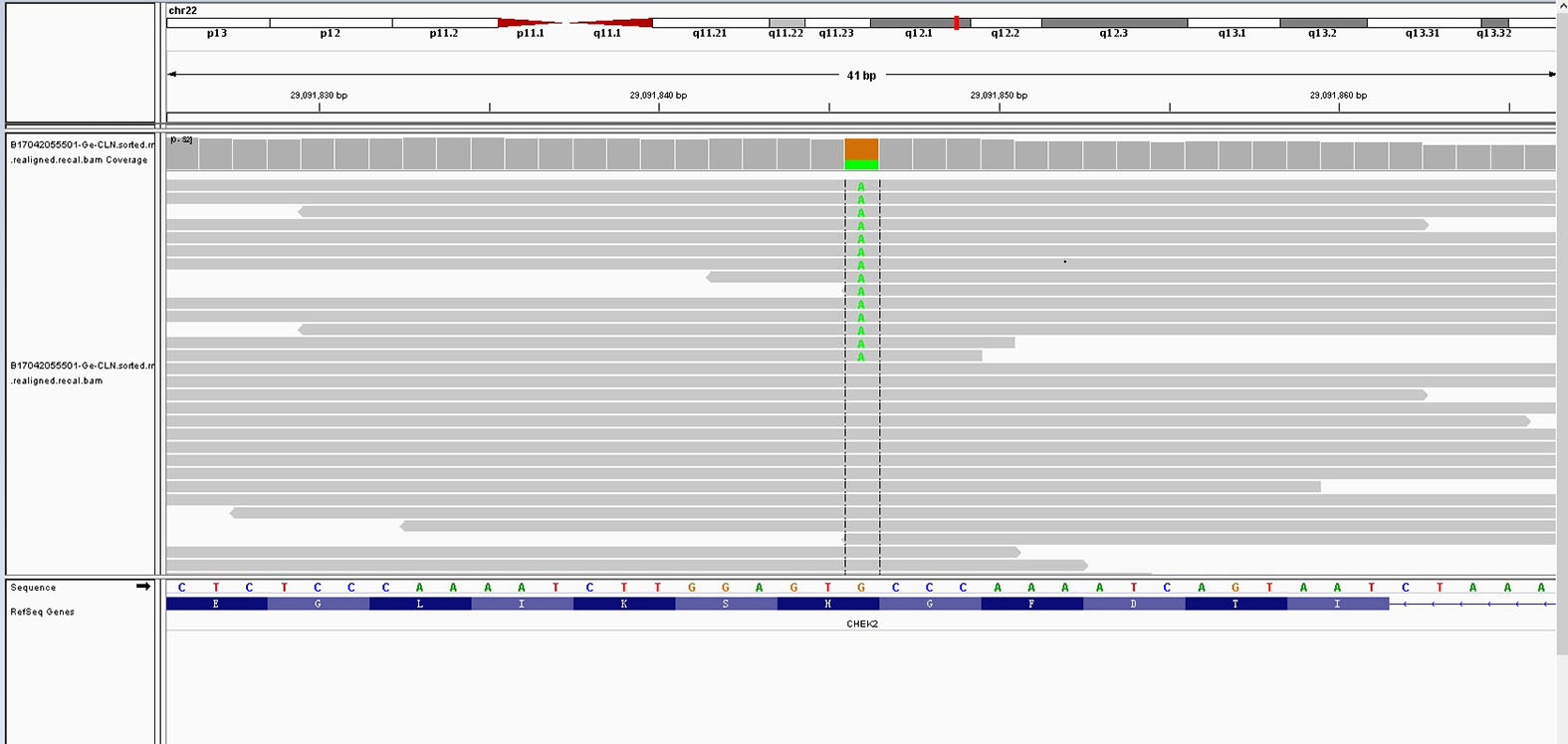

Supplement: Supplementary file 2 — Figure S2 Cell‐cycle‐checkpoint kinase 2 (CHEK2) is located on the long (q) arm of chromosome 22. p.H371Y germline mutation is caused by single base permutation in exon 11 of the CHEK2 gene. [file JOG-48-266-s001.jpg]
